# Diagnostic characteristics of 11 formulae for calculating corrected flow time as measured by a wearable Doppler patch

**DOI:** 10.1186/s40635-020-00339-7

**Published:** 2020-09-17

**Authors:** Jon-Émile S. Kenny, Igor Barjaktarevic, David C. Mackenzie, Andrew M. Eibl, Matthew Parrotta, Bradley F. Long, Joseph K. Eibl

**Affiliations:** 1 Health Sciences North Research Institute, Sudbury, ON P3E 2H2 Canada; 2 Division of Pulmonary and Critical Care, Department of Medicine, David Geffen School of Medicine at UCLA, Los Angeles, CA USA; 3 Department of Emergency Medicine, Maine Medical Center, Portland, ME USA; 4 Tufts University School of Medicine, Boston, MA USA; 5 Northern Ontario School of Medicine, Sudbury, ON Canada

**Keywords:** Carotid artery, Corrected flow time, Fluid responsiveness, Hemodynamics, Stroke volume

## Abstract

**Background:**

Change of the corrected flow time (Ftc) is a surrogate for tracking stroke volume (SV) in the intensive care unit. Multiple Ftc equations have been proposed; many have not had their diagnostic characteristics for detecting SV change reported. Further, little is known about the inherent Ftc variability induced by the respiratory cycle.

**Materials and methods:**

Using a wearable Doppler ultrasound patch, we studied the clinical performance of 11 Ftc equations to detect a 10% change in SV measured by non-invasive pulse contour analysis; 26 healthy volunteers performed a standardized cardiac preload modifying maneuver.

**Results:**

One hundred changes in cardiac preload and 3890 carotid beats were analyzed. Most of the 11 Ftc equations studied had similar diagnostic attributes. Wodeys’ and Chambers’ formulae had identical results; a 2% change in Ftc detected a 10% change in SV with a sensitivity and specificity of 96% and 93%, respectively. Similarly, a 3% change in Ftc calculated by Bazett’s formula displayed a sensitivity and specificity of 91% and 93%. Ftc_Wodey_ had 100% concordance and an *R*^2^ of 0.75 with change in SV; these values were 99%, 0.76 and 98%, 0.71 for Ftc_Chambers_ and Ftc_Bazetts_, respectively. As an exploratory analysis, we studied 3335 carotid beats for the dispersion of Ftc during quiet breathing using the equations of Wodey and Bazett. The coefficient of variation of Ftc during quiet breathing for these formulae were 0.06 and 0.07, respectively.

**Conclusions:**

Most of the 11 different equations used to calculate carotid artery Ftc from a wearable Doppler ultrasound patch had similar thresholds and abilities to detect SV change in healthy volunteers. Variation in Ftc induced by the respiratory cycle is important; measuring a clinically significant change in Ftc with statistical confidence requires a large sample of beats.

## Background

Change in the duration of systole has been used as an accurate surrogate for stroke volume (SV) in the critically ill, chronically ill, and in healthy volunteers [1–7]. Indeed, previous animal work employing isolated, supported heart preparations has shown that when heart rate and blood pressure are controlled, the duration of systole is directly related to SV [8–10]. Moreover, the direct relationship between systolic duration and SV was reaffirmed in humans using both indicator-dye dilution [11] and pressure gradient techniques [12]. Yet, the length of systole is also mediated by afterload [13, 14], contractility [15], and heart rate. Consequently, when using the change in systolic duration to predict change in SV, an underlying assumption is that afterload, contractility, and heart rate remain constant.

Constant heart rate may be achieved mathematically by calculating the corrected flow time (Ftc). Numerous Ftc formulae have been proposed and fall generally into linear [16–23] and non-linear [24–26] varieties. Furthermore, these formulae may be categorized by whether they correct for electrical systole (i.e., the duration from the onset of the Q wave to the end of the T wave) or mechanical systole (i.e., the duration from the upstroke of the pulse to the closure of the aortic valve).

**Fig. 1:**
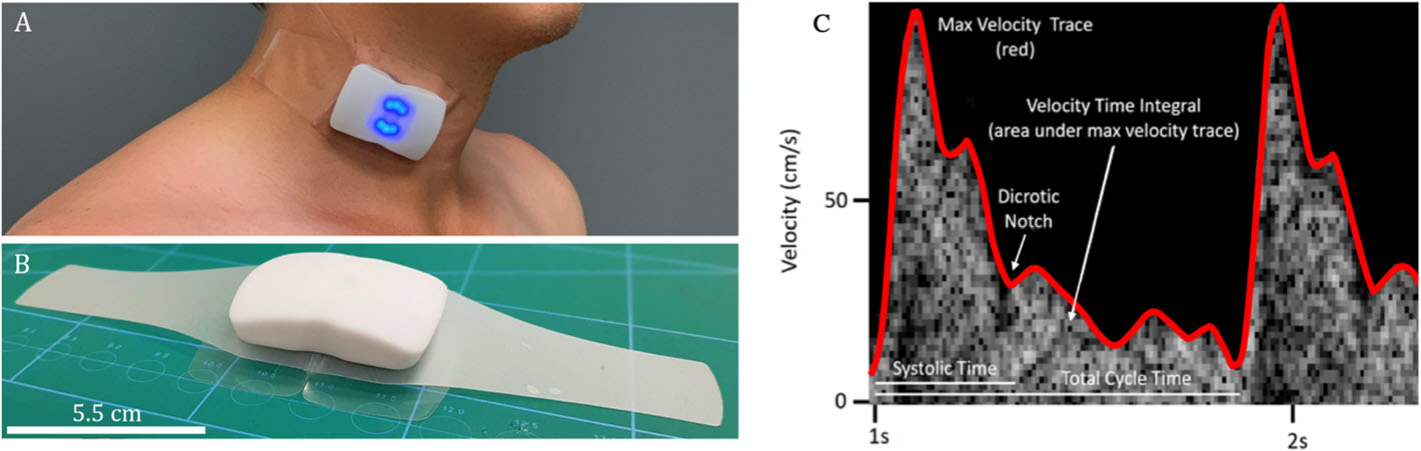
The wireless Doppler ultrasound patch. **a** Wireless, hands-free Doppler patch placed over common carotid artery of a healthy volunteer. **b** Scaled image of the Doppler patch. **c** Metrics obtained from wearable Doppler

Using a wearable Doppler ultrasound patch (Fig. 1) [27, 28], we studied 11 Ftc formulae for their ability to detect a 10% change in SV in healthy volunteers. Five of the Ftc formulae were initially derived to correct mechanical systole, while the remaining 6 were proposed to correct electrical systole for heart rate. Given that mechanical systole has better correlation with SV than electrical systole [12], we hypothesized that mechanical Ftc formulae would better detect SV change than those derived to correct electrical systole. Using the ultrasound patch capable of recording carotid Doppler spectra continuously, we evaluated the dispersion of Ftc during quiet respiration calculated by 2 Ftc formulae, as an exploratory analysis.

## Materials and methods

### *Clinical setting*

We recruited 26 healthy, adult volunteers. Exclusion criteria were known cardiovascular history and/or taking regular cardiovascular medications. The procedures followed were in accord with the ethical standards of the committee on human experimentation at our institution. Written and informed consent was obtained for all subjects, and the study was approved by the Research Ethics Board of Health Sciences North.

### Preload modifying maneuver

We have used the stand-squat-stand (SSS) maneuver previously as a reliable means to increase and decrease cardiac preload in healthy volunteers [27–30]. Of the 26 volunteers, 11 performed the SSS protocol as follows: 60 s of quiet standing followed by 60 s of passive squat and a subsequent 60 s return-to-stand; each subject performed the protocol in duplicate separated by at least 2 min of rest. Eight of the 26 volunteers performed the SSS in a manner identical to the first, however, rather than 60 s, each stand, squat, and return-to-stand lasted 72 s; this SSS protocol was performed in triplicate with at least 2 min of rest between maneuvers. Due to time constraints in the physiology lab, two subjects were unable to perform all three maneuvers. The remaining volunteers performed the SSS protocol once, in 40-s intervals. The reason for the differences in duration were that we have studied multiple SV monitors with different update times, as described previously [28]. In this investigation, we have restricted our results to non-invasive pulse contour analysis as the gold standard.

### Stroke volume monitoring

In all protocols, the Clearsight® (Edwards Lifesciences; Irvine, CA, USA) was applied to the subject in the standing position. Briefly, Clearsight® is a US Food and Drug Administration (FDA) approved non-invasive SV monitor that uses a “volume clamp” method to transduce the digital artery waveform. Using an algorithm, the digital artery waveform is transformed into a brachial artery waveform and then analyzed using pulse contour analysis to provide SV every 20 s [31]. A number of studies have evaluated the ability of Clearsight® to track changes in cardiac output with agreement values ranging between 84 and 100% compared to a gold standard [31]. The protocol did not begin until there was adequate Clearsight® signal as measured by the Physiocal calibration metric (i.e., ≥ 50). The third digit was used in all volunteers as recommended by the manufacturer and all subjects’ arms remained passively extended throughout the protocol so as not to change upper extremity arterial resistance and all subjects maintained normal, quiet tidal respiration during the maneuver. We compared baseline (T1) to peak squat (T2) as previously described [28]. Accordingly, the positional change from T1 to T2 increased cardiac preload. Subsequently, T2 was compared to repeat stand (T3); the change from T2 to T3 decreased cardiac preload.

### Corrected carotid flow time monitoring

A US FDA-approved wearable carotid Doppler patch (Flosonics Medical, Sudbury, ON, Canada) was placed by palpation over the carotid artery below the angle of the jaw in an effort to ensure Doppler sampling below the bifurcation of the common carotid artery. When an audible Doppler shift was heard and a Doppler spectrum consistent with the common carotid artery visualized, the Doppler patch was adhered in place. The maximum velocity of the Doppler waveforms was automatically traced using an algorithm based on the approach described by Li and colleagues [32]. The automated maximum velocity was used to determine the duration of systole from the systolic velocity upstroke to the dicrotic notch (i.e., systolic flow time) (Fig. 1c). The duration of systole in seconds was used to calculate the Ftc using 11 equations grouped by their initial derivation (Table 1). All Ftc equations were solved using a reference rate of 60 bpm as previously described [16, 33].

**Table 1:** Diagnostic characteristics of 11 Ftc formulae

	Thresh.	Sens.	Spec.	Conc.	R^2^
Mechanical Equations					
Ftc_Wodey_ = systolic time + 0.00129 (HR − 60) ^20^	2%	96%	93%	100%	0.75
Ftc_Weissler_ = systolic time + 0.0016 (HR − 60) ^11^	3%	91%	93%	98%	0.71
Ftc_Gobbato_ = systolic time + 0.00192 (HR − 60) ^18^	3%	91%	93%	95%	0.66
Ftc_Penati_ = systolic time + 0.00122 (HR − 60) ^19^	2%	93%	93%	99%	0.75
Ftc_Lian_ = cycle time [0.709 x systolic time x (cycle time + 0.41)] ^26^	4%	78%	87%	89%	0.61
Electrical Equations					
${Ftc}_{Bazett}=\frac{systolic\kern0.17em time}{\sqrt{cycle}\; time}$^24^	3%	91%	93%	98%	0.71
${Ftc}_{Fredericia}=\frac{systolic\kern0.17em time}{\sqrt[3]{cycle}\; time}$^25^	2%	87%	85%	90%	0.55
Ftc_Hodges_ = systolic time + 0.00175 (HR − 60) ^16^	3%	91%	93%	97%	0.68
Ftc_Framingham_ = systolic time + 0.154 (1.0 − cycle time) ^21^	2%	93%	93%	98%	0.73
Ftc_Adams_ = systolic time + 0.1464 (1.0 − cycle time) ^22^	2%	93%	93%	98%	0.74
Ftc_Chambers_ = systolic time + 0.127 (1.0 − cycle time) ^23^	2%	96%	93%	99%	0.76

*Thresh.* represents optimal threshold change in corrected flow time. *Sens.* is sensitivity (%) and *spec.* is specificity (%) for detecting a 10% change in stroke volume. *Conc.* is concordance rate (%) and *R*^2^ is the regression coefficient from the four-quadrant plot

### *Statistical analysis*

Values obtained for stroke volume during the preload modifying maneuvers were compared using a two-tailed Student’s *t* test. To evaluate the ability of Ftc formulae to track changes in SV values during the preload modifying maneuver, we computed four-quadrant plots and performed a concordance analysis [34]. The four-quadrant plot shows the relationship between changes in Ftc (*y*-axis) and changes in SV (*x*-axis) in a scatter plot. We defined a 10% change exclusion zone at the center of the plot to exclude changes in SV which are not clinically relevant. Based on the data points outside the exclusion zone, we calculated the concordance rate as the proportion (percentage) of concordant data pairs to all data pairs for each Ftc equation. As well, we quantified the sensitivity and specificity of changes in Ftc calculated by all Ftc formulae to detect significant changes in SV (> 10%) using all 100 preload changes.

Finally, as an exploratory analysis, we chose Wodeys’ and Bazetts’ equations as examples of linear and non-linear means to correct the duration of systole for heart rate and calculated the coefficient of variation (CV) and the least significant change (CV × 1.96 × √2) during quiet breathing in all subjects. The CV was calculated as the standard deviation of the Ftc relative to the mean Ftc for the entirety of all 50 initial quiet stands; then, an average of all 100 baseline CVs is reported.

## Results

The study was performed without any complication and hemodynamic parameters including blood pressure and heart rate remained within normal physiological ranges throughout the testing for all volunteers. The average age of the volunteers was 33 and 26% were females. The average body mass index was 23.5. In all subjects, audible Doppler and common carotid spectra were obtained and all subjects had adequate SV signals from the pulse contour analysis device.

### Effect of preload modification on hemodynamics

There were 100 changes in preload (i.e., 50 increase and 50 decrease) across the 26 subjects. Stroke volume rose in 50/50 protocols from T1 to T2; on average, SV increased by 21.9 mL or 25% (*p* < 0.0001). The 95% confidence interval (CI) for change in SV from T1 to T2 was + 21.5% to + 28.5%. Conversely, SV fell in 50/50 protocols from T2 to T3; the mean fall in SV on return-to-stand was 15.4 mL or − 17% (*p* < 0.0001) with a 95% CI of − 15.3 to − 20.3%. These results are congruent with our previous findings [27, 28]. There was no statistically significant difference in stroke volume change when comparing the three different timing protocols. Mean arterial blood pressure rose significantly from T1 to T2 from 98 mmHg to 104 mmHg (*p* < 0.001) and fell significantly from T2 to T3 from 104 to 99 mmHg (*p* < 0.001). We calculated arterial elastance (Ea) as (0.9 x systolic blood pressure)/stroke volume. On average, from T1 to T2, Ea fell from 1.40 to 1.24 mmHg/mL (*p* < 0.01) and then rose again to 1.40 at T3 (*p* < 0.01). Heart rate did not change significantly between T1, T2, and T3 at 80, 78, and 80 bpm, respectively.

### Diagnostic characteristics of Ftc formulae

**Fig. 2:**
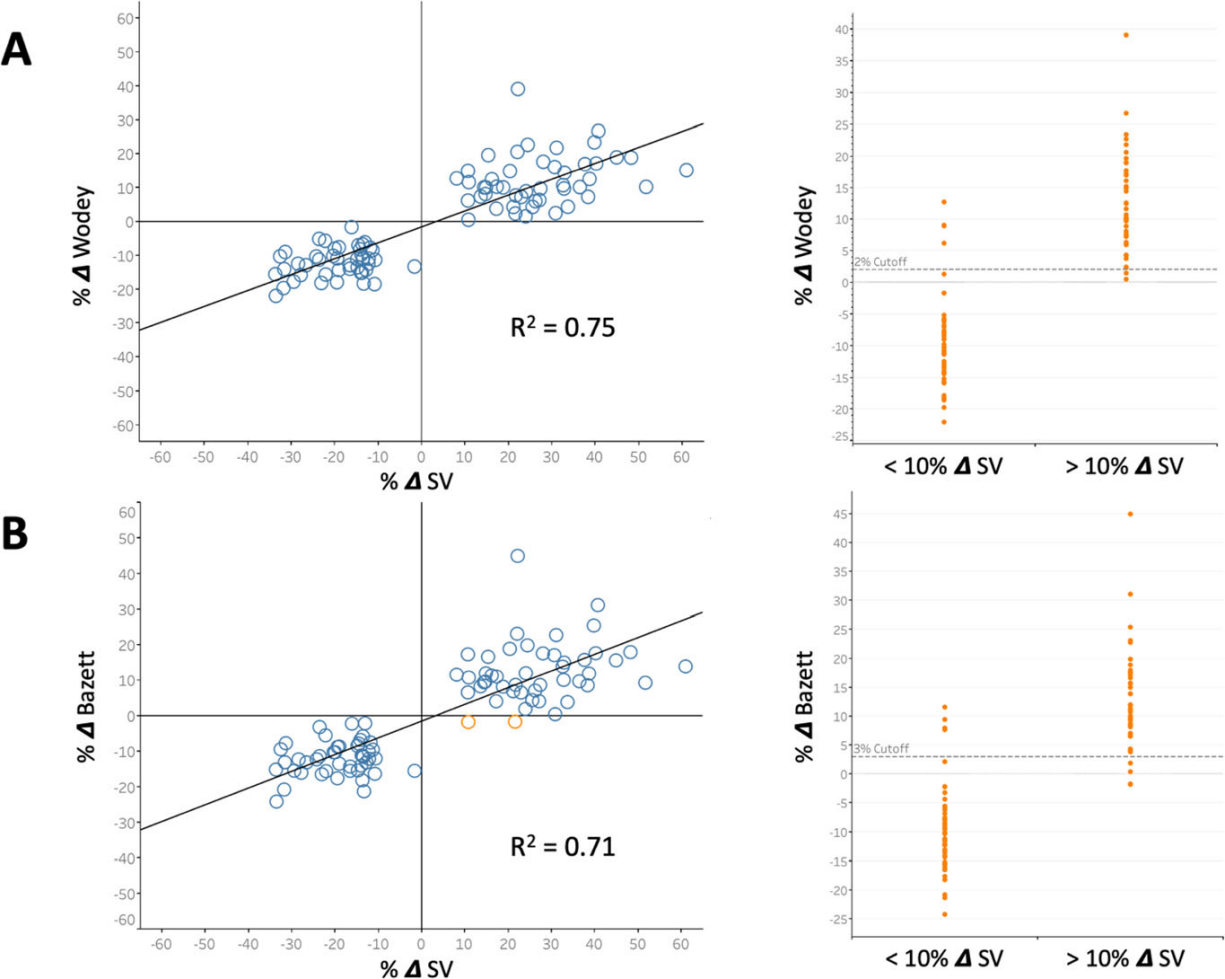
Diagnostic characteristics of 2 commonly encountered Ftc equations. All 100 preload changes are plotted in all graphs. **a** Represents the change in Wodey’s formula with the four-quadrant plot on the left and sensitivity-specificity analysis on right. A 2% cut-off provides the best discrimination. **b** The same data for Bazett’s formula; open orange points represent discordant data on the four-quadrant plot. A 3% threshold is the optimal discrimination for detecting 10% change in SV. SV is stroke volume; delta is change

In total, 3890 carotid beats were analyzed between T1, T2, and T3 in all volunteers. Table 1 summarizes best diagnostic threshold for detecting a 10% change in SV as well as the sensitivity, specificity, concordance, and regression coefficients of all 11 Ftc equations. Figure 2 shows the four-quadrant plot, regression coefficient as well as sensitivity, specificity analysis for Wodeys’ and Bazetts’ equations as these are commonly referenced equations in the literature. All 100 preload changes are plotted; for both equations on the four-quadrant plot, 8 data points were removed from the zone of exclusion. The area under the receiver operator curve (AUC) for Ftc_Wodey_ and Ftc_Bazett_ were 0.97 and 0.96, respectively. To compare the regressions between the equations, an *F*-test was performed with Ftc_Wodey_ as the reference equation. Compared to Ftc_Wodey_, only Ftc_Lian_ and Ftc_Fridericia_ had statistically significant variance.

### Coefficient of variation of Ftc during quiet breathing

**Fig. 3:**
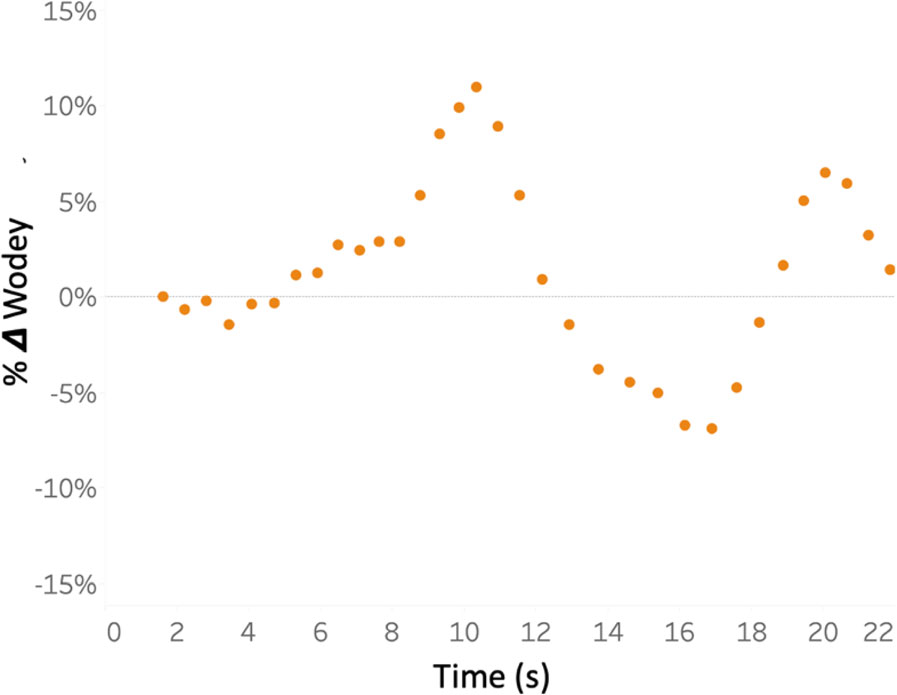
Respiratory variation in a representative subject. The percentage change in Ftc Wodey about the mean (*y*-axis) is illustrated for a single subject during 20 s (*x*-axis) of quiet standing

A total of 3335 carotid beats were evaluated during quiet breathing across all subjects during initial stand. A Kolmogorov-Smirnov test revealed that the distribution of Ftc during quite breathing was normal. The mean (± standard deviation) of Ftc by Wodeys’ and Bazetts’ equations was 0.286 s (± 0.0179 s) and 0.299 s (± 0.0299 s), respectively. The coefficients of variation for Ftc_Wodey_ and Ftc_Bazett_ during quiet respiration were 0.06 and 0.07, respectively. The least significant change for Ftc_Wodey_ and Ftc_Bazett_ were 0.17 and 0.19, respectively. Figure 3 is a representative example of respiratory variation in Ftc_Wodey_ from a single subject during 20 s of standing.

## Discussion

We report the diagnostic characteristics of 11 equations used to correct the duration of systole for heart rate as a method to predict change in stroke volume. Overall, the sensitivity, specificity, concordance, and regression coefficients were very similar for all formulae studied save for the equations of Lian and Fridericia. Both of these equations showed statistically significant variance as compared to Ftc_Wodey_ as the reference. Excepting Ftc_Lian_ and Ftc_Fridericia_, we observed no discernable difference between equations that were originally solved to correct electrical versus mechanical systole for heart rate. Ftc_Wodey_, Ftc_Penati_, and Ftc_Chambers_ are all linear regressions with nearly identical correction coefficients for heart rate. Consequently, despite the former two being initially derived to correct mechanical systole and the latter electrical systole, their diagnostic attributes were on par.

Two commonly used formulae are those of Wodey [1] and Bazett [2]. We found that the optimal threshold to detect a 10% change in SV using Ftc_Wodey_ was 2%. Similarly, 3% was the best discriminator for Ftc_Bazett_. These cut-offs identified a 10% difference in SV with a sensitivity and specificity of 96% and 93% for Wodey and 91% and 93% for Bazett (AUC 0.97 and 0.96, respectively). That Ftc_Wodey_ and Ftc_Bazett_ do not differ as surrogates for SV change is compatible with the findings of Mohammadinejad and Nossein-Nejad [35] who studied Ftc in 93 patients undergoing dialysis and 142 healthy volunteers during a passive leg raise (PLR).

Interestingly, the average standing baseline value of Ftc_Wodey_ in our study was 286 ms; thus, a threshold of 2% represents an absolute change of 6 ms. Notably, 7 ms was the optimal diagnostic cut-off identified by our group in patients with undifferentiated shock [1]. As well, our threshold is similar to the average absolute change in Ftc_Wodey_ following PLR in 142 healthy subjects [7]. Given that a 2% difference may be challenging to detect at the bedside, we performed an exploratory analysis to quantify variability in Ftc values at resting baseline. Because we measured the carotid spectra using a wearable Doppler patch, we assessed 3335 carotid beats during quiet standing across all 26 subjects; with this, we found the coefficient of variation for Ftc_Wodey_ and Ftc_Bazett_ to be 0.06 and 0.07, respectively. Thus, 95% of all carotid beats in resting volunteers fluctuate about the Ftc mean by ± 12–14% secondary only to variation imparted by the respiratory cycle. Given this intrinsic variation, detecting a 2% change in Ftc would require sampling 140 carotid beats both before and after an intervention in a single subject (assuming a type I error of 0.05 and power of 0.80) [36]. If the Ftc threshold were increased to 5% or 10%, then 22 or 5 beats both before and after an intervention would need to be sampled, respectively, to detect a change with statistical confidence. This may be challenging with traditional hand-held Doppler systems because quantifying the Ftc of multiple beats in succession is time-consuming at the bedside. Importantly, the variation described above accounts only for the respiratory cycle and does not include human factors [37]. Thus, the number of Doppler beats captured and measured in addition to human factors may be important arbiters of conflicting data [38].

Our current findings are consistent with our earlier data using an identical paradigm [27]. We previously found an excellent sensitivity and specificity for detecting a 10% change in SV using a 4% diagnostic threshold for change in Ftc_Wodey_. This updated analysis, with more subjects and data points, revealed an improved regression coefficient and equally good receiver operator characteristics, but with a slightly lower threshold for Ftc_Wodey_. The reason for this small difference may be due to inherent variability and sampling described above. Nevertheless, given the nature of the wearable Doppler patch, our current regression analyses are based upon 3890 heart beats, many times greater than most clinical studies using hand-held duplex Doppler systems.

The clinical implications of our data are that the change in corrected flow time from the carotid artery predicts changes in stroke volume, as previously reported in critically ill patients [1, 4]. For example, when a patient has signs of tissue hypoperfusion and intravenous fluids are considered, performing a passive leg raise with continuous Doppler monitoring can inform the clinician as to whether or not both ventricles will respond favorably to preload. Thus, a wireless, wearable Doppler ultrasound frees clinicians’ hands and continuously informs of the patient’s functional hemodynamic state. Such a device may be particularly useful early in care, for example, in the emergency department, or in the operating theatre because of ease-of-access to a patient’s neck. Further, our data show that independent of user variability, inherent variation in the Doppler trace induced by respiration prevents accurate comparison before and after an intervention if only a small sample of beats is analyzed. Therefore, the clinical value of an adherent Doppler may be both time-saving and convenience for the clinician but it may also limit user variability as well as capture, analyze, and account for inherent, physiological variation.

Our study has several limitations. First, we studied healthy volunteers with heart rates ranging between 42 and 155 bpm during preload modification; while this spectrum of heart rates is clinically acceptable, the bradycardia and tachycardia observed were typically transient, and we may have under-sampled extreme heart rates. This is especially important because non-linear equations like Bazett’s may be less accurate with tachycardia. Second, the volume clamp technique used to measure SV is limited when there is peripheral vasoconstriction (e.g., Raynaud’s syndrome, vasopressors) [31]. As none of our volunteers had peripheral vasoconstriction, the volume clamp method can provide reliable estimates of changing stroke volume [31]. While we have shown that changing SV, measured by volume clamp, is also tracked by Doppler of the descending aorta and bioreactance technologies [28], volume clamp is an indirect measure and may not be valid in surgical patients and the critically ill as compared to thermodilution [39, 40]. The advantages of the volume clamp method are that it is non-invasive and non-operator dependent. Third, we did not perform fluid challenges. Future studies are needed to investigate the ability of the Doppler patch to predict fluid responsiveness before fluid administration. Fourth, our data cannot account for changes in afterload and contractility as mediators of systolic time. Nevertheless, previous data employing both echocardiography and impedance cardiography found increased stroke volume on squatting without changes in contractility [29, 30]. The effect of squatting on afterload is mixed with previous data showing no change or decreased vascular resistance on squatting and minimal hemodynamic impact when squatting while supine or in a swimming pool [30, 41]. These data suggest that the primary mechanism of stroke volume augmentation on squat occurs by increased venous return when the gravitational gradient between the feet and heart is removed [41]; ultimately, this is physiologically akin to a passive leg raise. We found that the arterial elastance calculated from the non-invasive cardiac output monitor significantly fell from stand to squat (1.40 to 1.25 mmHg/mL) and then rose again on stand. As increased afterload in healthy volunteers has been observed to prolong left ventricular ejection time [13], one expects decreased elastance on squat to diminish ejection time, contrary to our findings. Nevertheless, given that the pulse contour analysis device calculates stroke volume from the pressure waveform, the arterial elastance calculation here may reflect mathematical coupling rather than a true change in arterial load. Additional study with full transthoracic echocardiography would help delineate specific mechanisms here. Finally, we did not account for differences between the internal and external carotid arteries. While others have found that blood flow in the internal carotid artery tracks changes in stroke volume [42], the congruence between internal and common carotid artery blood flow in the face of changing cardiac output is an avenue of future study.

## Conclusions

In conclusion, we describe the diagnostic characteristics of 11 Ftc equations using a wearable Doppler ultrasound patch during preload modifying maneuvers in healthy volunteers. We examined 3890 heart beats and found good correlation between change in Ftc and SV; Ftc_Wodey_ performed best overall. Additionally, small Ftc thresholds may be limited by Ftc variability induced by respiratory variation alone; a cut-off of 7 ms requires sampling over 100 beats before and after an intervention in a single subject to detect change with statistical confidence. The number of beats sampled could be reduced by raising the Ftc threshold, but this comes at a cost of sensitivity. Further evaluations in the emergency room, intensive care unit, and operating theatre are warranted.

## Data Availability

The datasets used and/or analyzed during the current study are available from the corresponding author on reasonable request.
